# Flexible All‐Inorganic Perovskite Photodetector with a Combined Soft‐Hard Layer Produced by Ligand Cross‐Linking

**DOI:** 10.1002/advs.202302005

**Published:** 2023-05-28

**Authors:** Tongyu Shi, Xi Chen, Rui He, Hao Huang, Xinru Yuan, Zhenyu Zhang, Jiahong Wang, Paul K. Chu, Xue‐Feng Yu

**Affiliations:** ^1^ Shenzhen Key Laboratory of Micro/Nano Biosensing Shenzhen Institute of Advanced Technology Chinese Academy of Sciences Shenzhen 518055 P. R. China; ^2^ University of Chinese Academy of Sciences Beijing 100049 P. R. China; ^3^ Hubei Three Gorges Laboratory Yichang Hubei 443007 P. R. China; ^4^ Department of Physics Department of Materials Science and Engineering and Department of Biomedical Engineering City University of Hong Kong Tat Chee Avenue, Kowloon Hong Kong P. R. China

**Keywords:** all‐inorganic perovskites, bendable, flexible photodetectors, ligands cross‐linking, nanocrystals, soft‐hard combination

## Abstract

Although perovskite nanocrystals have attracted considerable interests as emerging semiconductors in optoelectronic devices, design and fabrication of a deformable structure with high stability and flexibility while meeting the charge transport requirements remain a huge challenge. Herein, a combined soft‐hard strategy is demonstrated to fabricate intrinsically flexible all‐inorganic perovskite layers for photodetection via ligand cross‐linking. Perfluorodecyltrichlorosilane (FDTS) is employed as the capping ligand and passivating agent bound to the CsPbBr_3_ surface via Pb‐F and Br‐F interactions. The Si—Cl head groups of FDTS are hydrolyzed to produce Si—OH groups which subsequently condense to form the Si—O—Si network. The CsPbBr_3_@FDTS nanocrystals (NCs) are monodispersed cubes with an average particle size of 13.03 nm and exhibit excellent optical stability. Furthermore, the residual hydroxyl groups on the surface of the CsPbBr_3_@FDTS render the NCs tightly packed and cross‐linked to each other to form a dense and elastic CsPbBr_3_@FDTS film with soft and hard components. The photodetector based on the flexible CsPbBr_3_@FDTS film exhibits outstanding mechanical flexibility and robust stability after 5000 bending cycles.

## Graphical abstract

A combined soft‐hard strategy is demonstrated herein to fabricate intrinsically flexible all‐inorganic perovskite layers for photodetection via ligand cross‐linking. Perfluorodecyltrichlorosilane (FDTS) is employed as the capping ligand and passivating agent bound to the CsPbBr_3_ surface via Pb‐F and Br‐F interactions. The photodetector flexible CsPbB_r3_@FDTS film exhibits outstanding mechanical flexibility and robust stability after 5000 bending cycles.

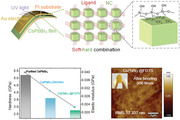

## 1 Introduction

Flexible photodetectors have attracted increasing attention as crucial components in wearable electronics, foldable displays, and portable image sensors.^[^ ^1^ ^]^ Conventional semiconductors such as Si, GaN, SiC, and InGaAs are dominating the photodetector market for the ultraviolet (UV) to near‐infrared spectral range.^[^ ^2^ ^]^ Nevertheless, traditional detectors composed of these semiconductors have a planar and rigid structure and cannot meet the stringent requirements of high‐performance flexible and deformable devices.^[^ ^3^ ^]^ Besides, the growth of these bulk materials and device fabrication typically involves a high temperature, thus the process is costly and complicated.^[^ ^4^ ^]^ The key challenge for flexible photodetectors is how to maintain the favorable photoelectric attributes of the materials while making them compatible with deformable substrates and meeting the simple and low‐cost manufacturing criteria.

Colloidal nanocrystals (NCs) are possible candidates for flexible devices by combining size‐dependent tunable optoelectronics with low‐temperature solution processing.^[^ ^5^ ^]^ In particular, all‐inorganic perovskites have attracted considerable interests as emerging semiconductors on account of the large absorption coefficient, high carrier mobility, and long carrier diffusion lengths.^[^ ^6^ ^]^ In fact, all‐inorganic perovskite NCs and flexible substrates can be integrated by spraying, spin coating, and drop casting to simplify the production process and reduce the cost.^[^ ^7^ ^]^ Specifically, van der Waals forces between ligands have been shown to bode well for the assembly of NCs to form films.^[^ ^8^ ^]^ However, small molecule ligands which stabilize the particle surface merely by coordination cannot sufficiently block surrounding moisture.^[^ ^9^ ^]^ Although the mechanical properties and stability can be improved by combining nanoparticles with polymers, the embedded nanoparticles suffer from the low volume fraction, poor dispersion in the polymer matrix, and an inert insulating coating.^[^ ^10^ ^]^ Hence, there is still a crucial need for a deformable structure that combines stability and high flexibility as well meets the charge transport requirements of photodetectors.

In this work, we design a stable flexible CsPbBr_3_@FDTS photosensitive layer with a combined soft‐hard structure based on the network skeleton formed by ligand cross‐linking at the perovskite NCs surface. 1H,1H,2H,2H‐perfluorodecyltrichlorosilane (FDTS), a fluorosilane of the F_3_C(CF_2_)_m_(CH_2_)_n_SiCl_3_ class, is employed as the capping ligand and passivating agent for CsPbBr_3_ perovskite via Pb‐F and Br‐F interactions. The Si—Cl groups of FDTS are converted into Si—OH driven by water molecules and further condense into Si—O—Si encapsulated on the surface of the perovskite NCs. Subsequently, the NCs are adopted as the building blocks to prepare a flexible perovskite film by self‐assembly and further condensation of Si—OH groups. Notably, the resistance to thermal and moisture improves significantly due to defect passivation and effective protection by the coating. Since the ultrathin modification layer of Si—O—Si network does not intercept the charge transport, we successfully fabricated the photosensitive layer with great photoelectric properties based on CsPbBr_3_@FDTS NCs. Moreover, the CsPbBr_3_@FDTS film with the combined soft‐hard structure has exceptional flexural strain resistance. Compared to the purified nanocrystal film (*E*≈5.2 GPa), the elastic modulus of the CsPbBr_3_@FDTS film decreases to 2.755 GPa illustrating significantly improved resistance to elastic deformation and better compatibility with flexible substrates under bending. As demonstration, the UV detector composed of the flexible CsPbBr_3_@FDTS film exhibits outstanding mechanical flexibility and robust stability after 5000 bending cycles.

## 2 Results and Discussion

**Figure 1:**
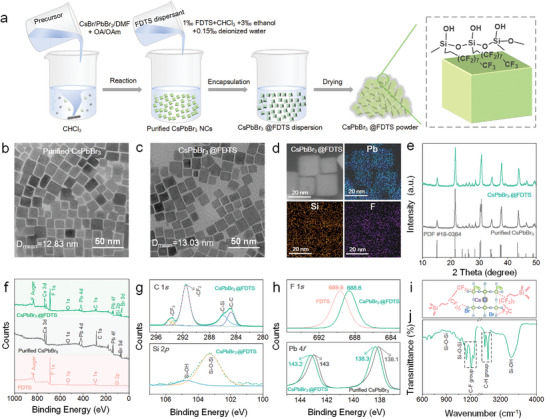
a) Schematic illustration of the CsPbBr_3_@FDTS NCs synthesis process. b,c) TEM images of b) Purified CsPbBr_3_ and c) CsPbBr_3_@FDTS NCs. d) EDS elemental maps of CsPbBr_3_@FDTS NCs. e) XRD spectra of the purified CsPbBr_3_ and CsPbBr_3_@FDTS NCs. f) XPS survey spectra of the purified CsPbBr_3,_ CsPbBr_3_@FDTS NCs, and FDTS. g) XPS C 1*s* and Si 2*p* spectra of CsPbBr_3_@FDTS. h) XPS F 1*s* spectra of CsPbBr_3_@FDTS and FDTS, Pb 4*f* spectra of CsPbBr_3_@FDTS, and purified CsPbBr_3_. i) Proposed structure and j) FTIR spectrum of CsPbBr_3_@FDTS.

The synthesis process of CsPbBr_3_@FDTS is illustrated in **Figure** **1**a; and Figure S1 (Supporting Information). First, the CsPbBr_3_ NCs are prepared by the ligand‐assisted reprecipitation method.^[^ ^11^ ^]^ After that, the as‐synthesized NCs are purified by centrifugation processes to remove dissociative organic ligands. As revealed by high‐resolution transmission electron microscopy (HR‐TEM), the purified CsPbBr_3_ NCs have a cubic shape (Figure 1b) with an average size of 12.83 nm (Figure S2a, Supporting Information). The CsPbBr_3_@FDTS NCs are produced by adding the FDTS dispersant (mixture of FDTS, chloroform, deionized water, and ethanol) into the solution of purified CsPbBr_3_ NCs under vigorous stirring. It is noted that these processes should be conducted under sealed conditions because moisture in air is adverse to driving the reaction uniformly. The obtained nanocrystals exhibit monodispersed cubes with an average size of 13.03 nm (Figure 1c; and Figure S2b, Supporting Information), which is close to that of purified CsPbBr_3_ NCs, indicating the formation of ultrathin modification layers.^[^ ^12^ ^]^ Both the purified CsPbBr_3_ and CsPbBr_3_@FDTS NCs have a lattice distance of 0.42 nm, corresponding to the (110) crystal plane of the monoclinic phase perovskite (Figure S3, Supporting Information). Compared to the purified CsPbBr_3_ NCs, elemental composition is detected from the perovskite surface by the energy dispersion spectroscopy (EDS) (Figure 1d; and Figure S4, Supporting Information). The X‐ray diffraction (XRD) patterns of the purified CsPbBr_3_ and CsPbBr_3_@FDTS NCs in Figure 1e can be indexed to CsPbBr_3_ (PDF#18‐0364), suggesting the coating does not change the perovskite structure.

To investigate the surface chemistry of CsPbBr_3_@FDTS NCs, X‐ray photoelectron spectroscopy (XPS) is performed. As shown in Figure 1f, F *1s* and Si 2*p* peaks are detected from the purified FDTS and CsPbBr_3_@FDTS NCs, while Pb, Cs, and Br are observed from purified CsPbBr_3_ and CsPbBr_3_@FDTS NCs, thus confirming synthesis of CsPbBr_3_ and introduction of FDTS. In the C 1*s* spectra of CsPbBr_3_@FDTS, the peaks at 284.8, 285.9, 291.5, and 293.5–294.2 eV correspond to the C—C, C—Si, —CF_2_ and —CF_3_ groups, respectively (Figure 1g).^[^ ^13^ ^]^ The high‐intensity —CF_2_ bond is consistent with the —(CH_2_)_2_(CF_2_)_7_CF_3_ chemical group of FDTS, demonstrating successful binding of the long‐chain fluoroalkyl group to the surface of CsPbBr_3_.^[^ ^14^ ^]^ The Si spectrum of CsPbBr_3_@FDTS can be divided into two peaks at 103.3 and 104.7 eV attributable to Si—OH and Si—O—Si, respectively.^[^ ^14^, ^15^ ^]^ The Si—OH peak is weaker than the Si—O—Si peak, probably due to condensation of most of the Si—OH to form the Si—O—Si structure. The interaction between FDTS and CsPbBr_3_ can also be confirmed by the chemical shifts in XPS measurement (Figure 1h). Compared to the purified CsPbBr_3_ and FDTS, the F 1*s* peak of CsPbBr_3_@FDTS decreases from 689.6 to 688.6 eV, while the Pb 4*f* and Br peaks (Figure S5, Supporting Information) shift to higher energies. These shifts can be attributed to the change in the chemical environment including the formation of halogen bonds (between F^−^ and Br^−^)^[^ ^16^ ^]^ and electronic interactions (betweenPb^2+^ and F^−^),^[^ ^17^ ^]^ and the probable binding scheme is proposed in Figure 1i.^[^ ^18^ ^]^

Fourier‐transform infrared spectroscopy (FTIR) is conducted and as shown in Figure 1j, the obvious stretching vibration peak of —OH at 3441 cm^−1^ of CsPbBr_3_@FDTS stems from hydrolysis of the Si—Cl head group of FDTS.^[^ ^19^ ^]^ The Si—O—Si vibration peak at 1070 cm^−1^ and Si—O symmetrical stretching peak at 897 cm^−1^ arise from condensation of Si—OH into the Si—O—Si structure.^[^ ^13^, ^20^ ^]^ The bands at 2922 and 2851 cm^−1^ are assigned to C—H bonds of the —(CH_2_)_2_(CF_2_)_7_CF_3_ groups^[^ ^13a^ ^]^ and those at 1148–1236 cm^−1^ can be ascribed to vibration of —CF_3_ and —CF_2_ functional groups.^[^ ^14^ ^]^

Based on the experimental results, the formation mechanism of the in situ network‐like coating of Si—O—Si on the CsPbBr_3_ surface is described in Scheme S1 (Supporting Information). FDTS first binds to the surface of CsPbBr_3_ NCs by Pb‐F and F‐Br coordination. In particular, the C—F chain is located on the inner side due to the affinity and interaction with the perovskite surface, and the outer Si—Cl groups are converted to S—OH driven by water molecules. Subsequently, the Si—OH groups condense to form the Si—O—Si network on the surface of CsPbBr_3_ NCs. Since hydrolysis of FDTS is vigorous and a trace amount of water vapor in the air is able to initiate the reaction, further control of the reaction uniformity is required (Figure S6, Supporting Information). The reaction rate can be regulated by controlling the amounts of H_2_O and ethanol in the dispersion, where water promotes the hydrolysis reaction and ethanol acts as an inhibitor (Figure S7, Supporting Information). The temperature also plays an important role in the reaction, as heating promotes condensation, which is not conducive to the formation of monodispersed particles. With the increase of FDTS addition, the obvious coating layer can be observed in the TEM images (Figure S8, Supporting Information). After coated with Si—O—Si network structure, the measured contact angle is about 76°, and the affinity for water is mainly caused by the residual Si—OH group on the surface (Figure S9, Supporting Information).

**Figure 2:**
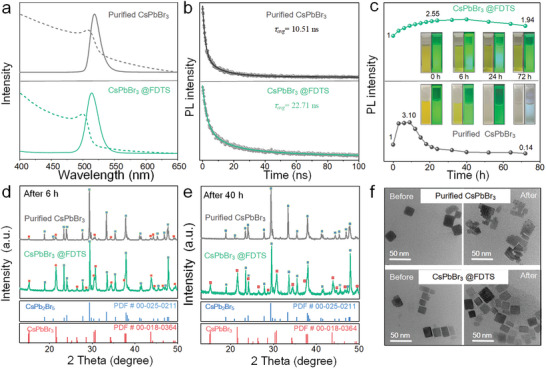
a,b) Absorption and PL spectra and fitted curves of the purified CsPbBr_3_ and CsPbBr_3_@FDTS NCs. c) Time‐dependent PL intensity of the NCs immersed in water. The insets are the optical images of the perovskite aqueous solution under light and UV illumination. d,e) XRD patterns of the purified CsPbBr_3_ and CsPbBr_3_@FDTS NCs after immersion in water for 6 and 40 h. f) TEM images of the purified CsPbBr_3_ and CsPbBr_3_@FDTS NCs before and after immersion in water for 40 h.

The optical properties of the CsPbBr_3_@FDTS NCs are presented in **Figure** **2**a; and Figure S10 (Supporting Information). After the FDTS treatment, the absorption and photoluminescence (PL) emission spectra show a slight blue shift, presumably caused by ion exchange between Br^−^ of CsPbBr_3_ and Cl^−^ of FDTS, and the enhanced PL intensity demonstrating the defects on the surface are effectively passivated. The time‐resolved photoluminescence (TRPL) spectrum of the perovskite NCs can be fitted by a bi‐exponential function with two decay components as shown in Table S1 (Supporting Information). The PL lifetimes of the CsPbBr_3_ NCs with the oleate‐olylamino ligand (CsPbBr_3_ OA/OAm), purified CsPbBr_3_ NCs, and CsPbBr_3_@FDTS are 12.31, 10.54, and 22.71 ns, respectively (Figure 2b; and Figure S11, Supporting Information). The significant increase in the CsPbBr_3_@FDTS short lifetime (*τ*_1_) and long lifetime (*τ*_2_) indicates that FDTS modification produces effective surface defect passivation.

To evaluate the resistance of CsPbBr_3_@FDTS NCs to water, the PL emissions are tracked as shown in Figure 2c. When the samples are immersed in water, the PL emission of purified CsPbBr_3_ exhibits a sharp increase (0–6 h), followed by rapid decline and almost complete quenching after 40 h. In contrast, the CsPbBr_3_@FDTS sample shows a slow increase and then a gradual decrease, notably maintaining highly luminescence even after 72 h. According to XRD, the CsPbBr_3_ NCs are converted into CsPb_2_Br_5_ in contact with water. As for the purified CsPbBr_3_ NCs without a protective layer to resist attack by water, a continuous phase transition occurs until the CsPbBr_3_ structure is destroyed. As for the CsPbBr_3_@FDTS NCs coated with Si—O—Si network, the partially exposed CsPbBr_3_ in the outer layer transforms into the CsPb_2_Br_5_ structure to form a relatively stable dual phase heterojunction which improves the PL performance and stability against hydrolytic degradation.^[^ ^21^ ^]^ The TEM images corroborate the better structural stability of the CsPbBr_3_@FDTS NCs as shown in Figure 2f. CsPbBr_3_@FDTS also shows more stable PL emission under harsh conditions such as 85 °C and 85% relative humidity (Figure S12, Supporting Information).

**Figure 3:**
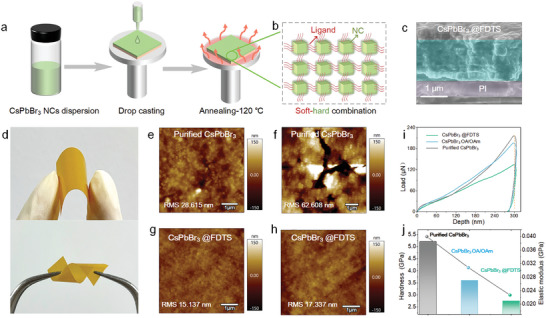
a) Schematic illustration of the CsPbBr_3_ films synthesis process. b) Illustration of the combined soft‐hard structure. c) Scanning electron microscope (SEM) image of the cross‐section of the CsPbBr_3_@FDTS film. d) Mechanical properties: distortion and flexure operation of CsPbBr_3_ films. AFM images of the purified CsPbBr_3_ film e) before and f) after deformation. AFM images of the purified CsPbBr_3_ film g) before and h) after deformation. i) Typical load‐displacement curves, j) elastic modulus and hardness values of the purified CsPbBr_3_, CsPbBr_3_@FDTS, and CsPbBr_3_ OA/OAm films.

Perovskite films are prepared on the polyimide (PI) substrate by the drop coating method as shown in **Figure** **3**a.^[^ ^22^ ^]^ Compared with the purified CsPbBr_3_ film with holes and small cracks, the CsPbBr_3_@FDTS film shows a uniform and dense surface (Figure S13, Supporting Information). The better homogeneity stems from that the residual Si—OH groups on the perovskite surface can drive the nanocrystals to be closely packed and cross‐linked to each other consequently forming an elastic film based on the soft‐hard combination (Figure 3b). As shown in Figure 3c, the thickness of the perovskite film is about 2 µm. The morphological changes of the CsPbBr_3_ NCs films caused by deformation are investigated (Figure 3d). As revealed by atomic force microscopy (AFM), the purified CsPbBr_3_ film has a different morphology after distortion and flexural operation showing obvious cracks and roughness increase from 28.615 to 62.608 nm (Figure 3e,f). On the contrary, the FDTS‐modified group shows an almost unchanged dense and uniform surface with a roughness of 17.337 nm, reflecting better resistance to deformation (Figure 3g,h). Nanoindentation experiments are performed to further evaluate the mechanical properties of the perovskite films (Figure 3i). The elastic modulus of the purified CsPbBr_3_ film is derived to be 5.207 GPa from the load‐displacement curve^[^ ^23^ ^]^ and it is significantly higher than that of the CsPbBr_3_ OA/OAm film (3.600 GPa), thus confirming the large influence of the organic ligands on the mechanical properties (Figure 3j). The elastic modulus and hardness of the CsPbBr_3_@FDTS film are 2.755 and 0.027 GPa, respectively, which are obviously lower than those of the purified CsPbBr_3_ and CsPbBr_3_ OA/OAm films thereby providing evidence of the positive role of the soft‐hard structure in the flexibility.

**Figure 4:**
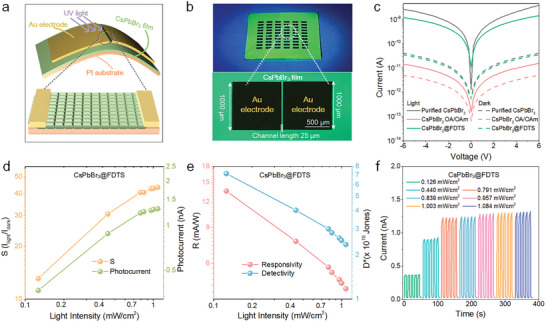
a) Schematic illustration and b) photographs of the photodetector under UV illumination. c) *I*–*V* curves of the purified CsPbBr_3_, CsPbBr_3_@FDTS, and CsPbBr_3_ OA/OAm based photodetectors. d) Photocurrent, switching ratio, e) responsivity and specific detectivity of the CsPbBr_3_@FDTS devices as a function of light intensity at a bias of 5 V. f) *I*–*T* characteristics of the CsPbBr_3_@FDTS devices under different illumination intensity at a bias of 5 V.

**Figure** **4**a shows the schematic illustration of the flexible photodetector consisting of the CsPbBr_3_ film as the photosensitive layer. The optical images of the photodetector arrays prepared on the PI substrate with the Au electrode channel length of 25 µm under illumination are depicted in Figure 4b. To investigate the optoelectronic properties of the perovskite photodetectors, the logarithmic current–voltage (*I*–*V*) curves in the dark and under 365 nm illumination are presented in Figure 4c. Different from the insulating effects of the OA/OAm ligands on charge transport, the perovskite coated Si–O–Si network maintains the charge carrier transfer characteristics. Besides, *I*–*V* curves with different light intensities are shown in Figure S14 (Supporting Information). It can be observed that the photocurrent (*I*_ph_ = *I*_light_‐*I*_dark_) and the switch ratio (*I*_light_/*I*_dark_) of CsPbBr_3_@FDTS photodetector increase when the light powder increases from 0.126 to 1.084 mW cm^−2^ at 5.0 V bias voltage (Figure 4d).

The responsivity (*R*) which reflects the response ability of the photodetector to illumination is expressed by *R* = *I*_ph_/(P·A), where P and A denote the light intensity and effective illumination area, respectively.^[^ ^2c^ ^]^ The specific detectivity (D*) representing the capability to detect the weakest light signals is expressed by D* = *R*/(2*qI*_dark_/*A*)^1/2^, where *q* is the elementary charge.^[^ ^24^ ^]^ The *R* and D* values of the CsPbBr_3_@FDTS photodetector under various illumination at a bias of 5 V exhibit a dependence on the light intensity, while the maximum values are 13.60 mA W^−1^ and 7.75×10^10^ Jones with the light intensity of 0.126 mW cm^−2^, respectively (Figure 4e). The performance of detectors fabricated by CsPbBr_3_@FDTS with different sizes and FDTS‐to‐NCs ratios is summarized in Table S2 (Supporting Information). The performances of the CsPbBr_3_@FDTS photodetector based on other purified CsPbBr_3_ device are shown in Figures S15 and S16 (Supporting Information). Moreover, Figure 4f; and Figure S17 (Supporting Information) show the current−time characteristics of the perovskite devices under different illumination intensities at a bias of 5.0 V. Compared to the drifting dark current baseline and continuously increasing photocurrent observed from the purified perovskite detector, the CsPbBr_3_@FDTS photodetector has better stability and reversibility under continuous light on/off switching.

**Figure 5:**
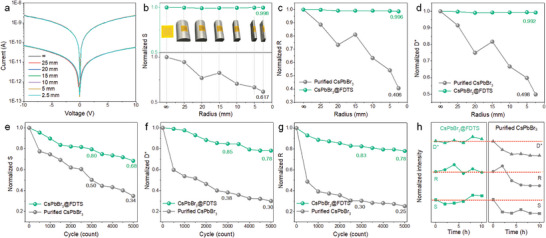
a) *I*–*V* curves of the CsPbBr_3_@FDTS based photodetector under different bending radii (laser illumination: 365 nm, 0.791 mW cm^−2^). Normalized b) switching ratios, c) responsivities, and d) specific detectivities of the purified CsPbBr_3_ and CsPbBr_3_@FDTS based photodetectors in different bending states with the inset showing the corresponding photographs of the device bent at different angles at a bias of 5 V. Normalized e) switching ratios, f) specific detectivities, and g) responsivities of the purified CsPbBr_3_ and CsPbBr_3_@FDTS based photodetectors under 5000 times of bending cycles at a bias of 5 V. h) Photodetection properties under damp and hot conditions (85 °C and relative humidity, 85%) at a bias of 5 V.

The mechanical stability of the CsPbBr_3_ photodetectors is assessed and presented in **Figure** **5**. Figure 5a; and Figure S18 (Supporting Information) show the *I*−*V* curves of the perovskite detectors versus bending radii upon 365 nm laser illumination (0.791 mW cm^−2^) under ambient conditions. As the bending radii decrease (inset of Figure 5b), the purified CsPbBr_3_ based photodetector shows declining performance and the calculated *S*, *R*, and D* decay are approximately half of the initial values for a radius of 2.5 mm (Figure 5b–d). On the contrary, the flexible CsPbBr_3_@FDTS photodetector shows no obvious loss in response to the photocurrent from a flat state to a radius of 2.5 mm, demonstrating significantly improved electrical stability and durability under bending. As shown in Table S3 (Supporting Information), the overall performance of the FDTS modified photodetector exhibits enhanced working stability with less current drift, satisfactory flexible cycling tests and competitive detection performance compared with other photodetectors.

After 3000 bending cycles, the performance index of CsPbBr_3_@FDTS flexible photodetector maintains over 80% of the initial values, while the calculated normalized *S*, D*, and *R* of the purified CsPbBr_3_ based device are 0.50, 0.38, and 0.30, respectively (Figure 5e–g). Even after 5000 bending cycles, the flexible CsPbBr_3_@FDTS photodetector delivers robust performance. The excellent mechanical stability can be attributed to the full release of bending stress through the CsPbBr_3_@FDTS film in which the combined soft‐hard structure plays a crucial role.^[^ ^25^ ^]^ As shown in Figure 5h, the CsPbBr_3_@FDTS‐based device exhibits negligible degradation and retains almost the initial performance after 10 h in the damp and hot environment (85 °C and relative humidity of 85%).

## 3 Conclusion

In conclusion, a combined soft‐hard strategy is demonstrated to fabricate intrinsically flexible layers for photodetection by ligand cross‐linking. Our results suggest that the FDTS ligands bind firmly to CsPbBr_3_ surface through Pb‐F and Br‐F interactions and provide effective surface passivation and protection. The introduced Si—OH groups cross‐link to form the Si—O—Si network and drive the assembly of the perovskite NCs to form the CsPbBr_3_@FDTS film, consequently producing a perovskite nanocrystal film with exceptional flexibility. The elastic modulus of CsPbBr_3_@FDTS film is 2.755 GPa which shows significantly improved resistance to elastic deformation and compatibility with the flexible substrate in the bending test. As a result, the UV detector composed of the flexible CsPbBr_3_@FDTS photosensitive layer has outstanding mechanical flexibility and robust stability as demonstrated by the bending test of 5000 cycles. We believe that our work provides a strategy to balance the stability and flexibility while simultaneously offering excellent charge transport boding well for optoelectronic devices, greatly extending the application of NCs in future optoelectronic devices.

## Conflict of Interest

The authors declare no conflict of interest.

## Supporting information

Supporting Information

## Data Availability

The data that support the findings of this study are available from the corresponding author upon reasonable request.
