# Supplementary material for: Flexible All‐Inorganic Perovskite Photodetector with a Combined Soft‐Hard Layer Produced by Ligand Cross‐Linking
Source: Adv Sci (Weinh). 2023 May 28;10(22):2302005. doi: 10.1002/advs.202302005 (PMC10401168; doi:10.1002/advs.202302005)
Supplement: Supplementary file 1 — Supporting Information [file ADVS-10-2302005-s001.pdf]

ADVANCED SCIENCE
Open Access

Supporting Information

for *Adv. Sci.*, DOI 10.1002/advs.202302005

Flexible All-Inorganic Perovskite Photodetector with a Combined Soft-Hard Layer Produced by Ligand Cross-Linking

Tongyu Shi, Xi Chen, Rui He, Hao Huang, Xinru Yuan, Zhenyu Zhang, Jiahong Wang, Paul K. Chu and Xue-Feng Yu**

Supporting Information

Flexible All-Inorganic Perovskite Photodetector with a Combined Soft-Hard Layer Produced by Ligand Cross-Linking

Tongyu Shi[1,2], Xi Chen[1,2], Rui He[1], Hao Huang[1], Xinru Yuan[1,2], Zhenyu Zhang[1], Jiahong Wang[1,2,3], Paul K. Chu[4], Xue-Feng Yu[1,2,3]**

METHOD

Materials. The main chemical reagents including cesium bromide (CsBr, Aladin, 99.9%), lead bromide ($PbBr_2$, Aladin, 99.0%), N,N-dimethylformamide (DMF, Aladin, 99.9%), oleylamine (OAm, Aladin, C18:80–90%), oleic acid (OA, Aladdin, AR), 1H,1H,2H,2H-Perfluorodecyltrichlorosilane (FDTS, Aladin, 96%), chloroform (Sinopharm, 99.0%), ethonal (Sinopharm, 99.8%), acetone (Sinopharm, 99.0%), gold (Zhongnuo Advanced Material (Beijing) Technology Co., Ltd, 99.99%) were used without purification, except chloroform which was dehydrated before use.

Synthesis of purified $CsPbBr_3$ NCs. The $CsPbBr_3$ NCs were prepared by the ligand-assisted reprecipitation method. Firstly, CsBr (0.4 mmol), $PbBr_2$ (0.4 mmol) were dissolved in DMF (10 mL). Subsequently, OAm (0.1 mL) and OA (1 mL) were added to stabilize the precursor solution until the powder was completely dissolved. 1 mL of the precursor solution was added quickly into chloroform (10 mL) under vigorous stirring, and strong green emission was observed immediately. After three times centrifugation (10,000 rpm, 10 min), purified $CsPbBr_3$ NCs can be obtained.

Synthesis of $CsPbBr_3$@FDTS NCs. The FDTS dispersant should be freshly prepared before use (10 mL chloroform, 30 μL ethanol, 1.5 μL deionized water and 10 μL FDTS). Then 1 mL chloroform and 1 mL FDTS dispersant were added into the redispersed purified $CsPbBr_3$ NCs chloroform solution (2 mL) in sequence with a 5 min interval. After stirring for 10 min, 1 mL chloroform and 1 mL FDTS dispersant were added again. The obtained colloid solution was centrifugated (10,000 rpm, 10 min) to get the $CsPbBr_3$@FDTS NCs, which were used for the TEM characterization (Figure

1c) and photodetector fabrication. After further drying at 50 °C, the $CsPbBr_3$@FDTS powders were used for other material characterizations and stability tests.

Preparation of the $CsPbBr_3$@FDTS flexible photodetector. The $CsPbBr_3$@FDTS NCs were redispersed in chloroform to form a 10 g/mL uniform dispersion. Polyimide (PI) membranes (2.5 cm × 2.5 cm) were cleaned sequentially in acetone, ethanol and deionized water. The $CsPbBr_3$@FDTS film was obtained by the drop-casting method, and then the gold electrodes (1 mm × 1 mm) were deposited on the composite film by thermal evaporation, and the channel length between the Au electrodes is 25 μm.

Characterization

The morphology of nanocrystals was examined by transmission electron microscopy (TEM, FEI Talos F200X) at 200 kV. The composition was determined by energy-dispersive X-ray spectroscopy (EDS) using the accessory manufactured by Oxford instruments. To characterize the structure, powder X-ray diffraction (XRD) was performed on a Rigaku Smartlab 3kW X-ray diffractometer with Cu K_α radiation (λ = 1.54056 Å, 40 kV, 30 mA, 10° min^{-1} from 10 to 60°). X-ray photoelectron spectroscopy (XPS) was carried out on the Thermo ESCALAB 250Xi system. The structure of the composites was analyzed by Fourier transform infrared spectroscopy (FTIR, Thermo Scientific, Nicolet iS60) with the KBr pellet method. The PL and TRPL properties were determined using a fluorescent spectrophotometer (Hitachi F-4600 and Edinburgh FLS-1000) at an excitation wavelength of 365 nm. The absorption spectra were obtained on the Lambda25 UV-Vis-NIR spectrophotometer. The morphology of the perovskite films was examined by scanning electron

microscopy (SEM, Zeiss GeminiSEM 300) and atomic force microscopy (AFM, Bruker, USA). The mechanical properties such as elastic modulus and hardness were detected by a Nanoindenter XP (Bruker Hysitron TI950). Electric and optoelectronic measurements of the fabricated devices were conducted in darkness and recorded by the PDA FS380 semiconductor analyzer.

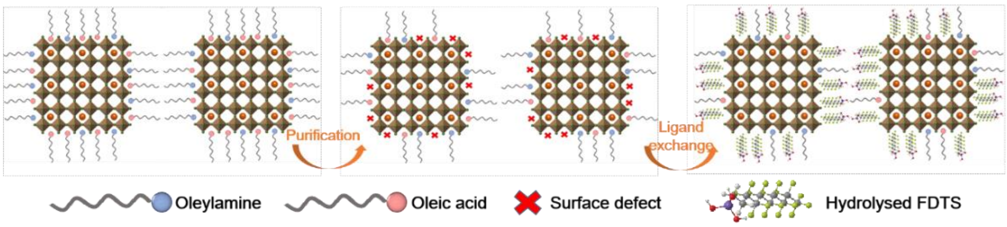

Figure S1. Schematic illustration of the $CsPbBr_3$@FDTS NCs synthesis process

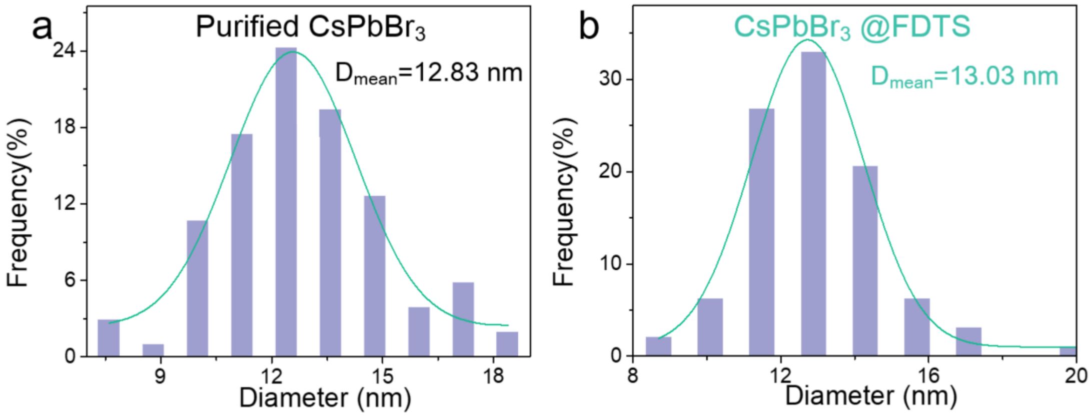

Figure S2. Particle size distributions: (a) purified $CsPbBr_3$ NCs and (b) $CsPbBr_3$@FDTS.

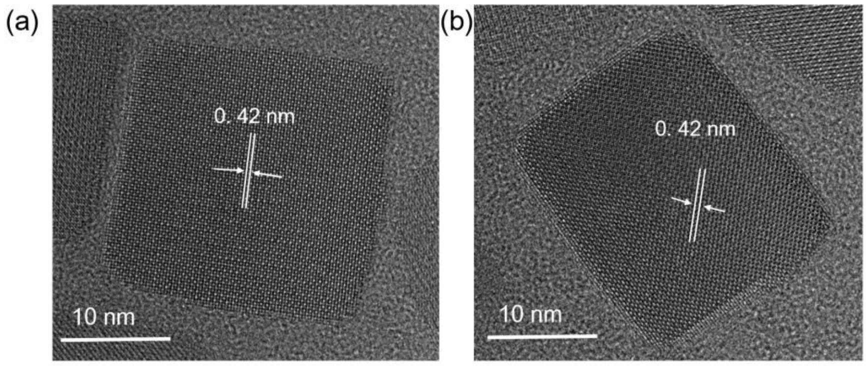

Figure S3. HRTEM images of (a) purified $CsPbBr_3$ and (b) $CsPbBr_3$@FDTS NCs

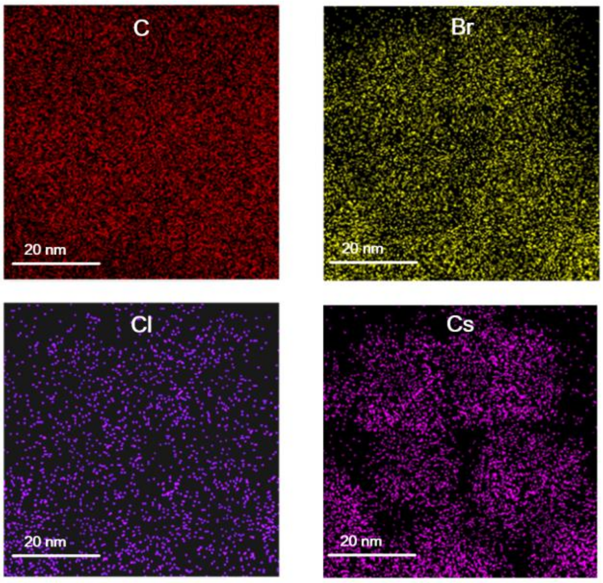

Figure S4. EDS elemental maps of $CsPbBr_3$@FDTS NCs.

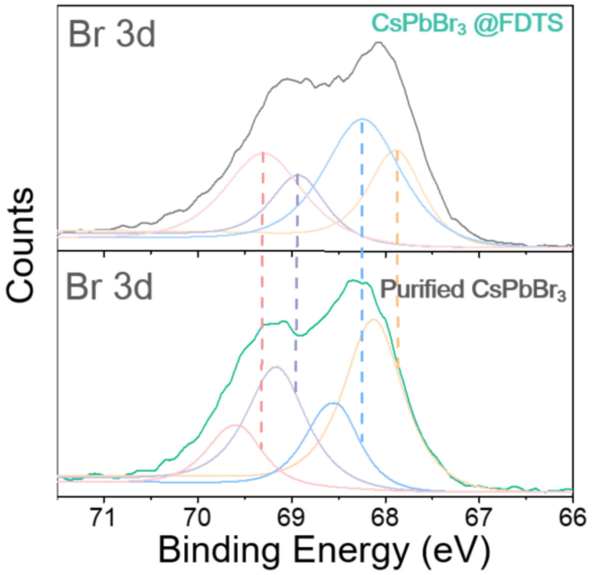

Figure S5. Br 3*d* XPS spectra of purified $CsPbBr_3$ NCs and $CsPbBr_3$@FDTS.

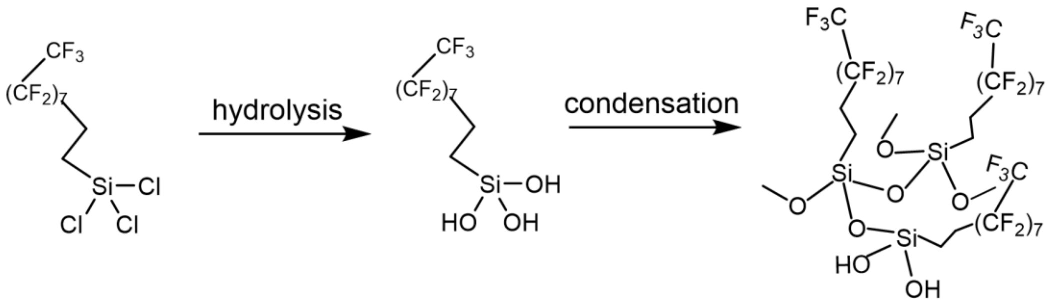

Scheme S1. Formation of the *in situ* network-like coating of Si-O-Si on $CsPbBr_3$.

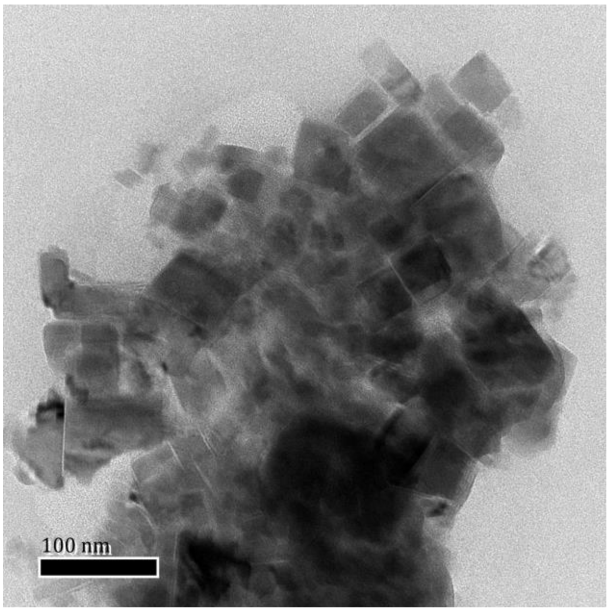

Figure S6. TEM image of $CsPbBr_3$@FDTS synthesized under ambient conditions (25 ℃ and relative humidity of 75%).

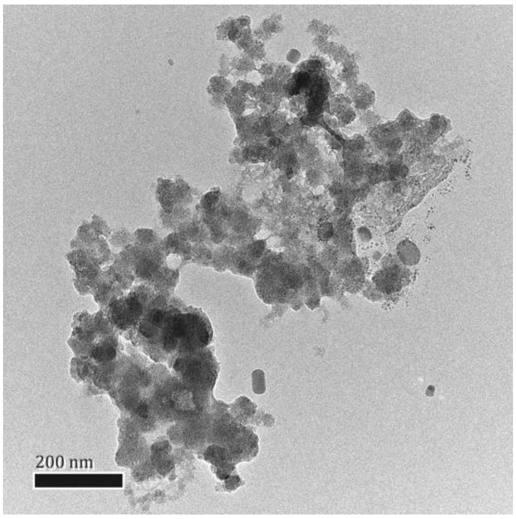

Figure S7. TEM image of $CsPbBr_3$@FDTS synthesized without ethanol addition in a sealed environment.

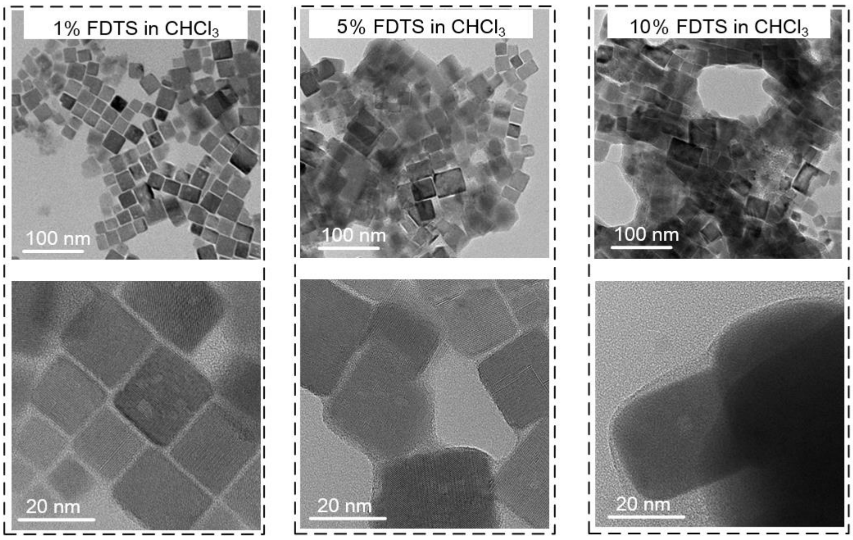

Figure S8. TEM images of the $CsPbBr_3$@FDTS NCs with different amount of FDTS addition

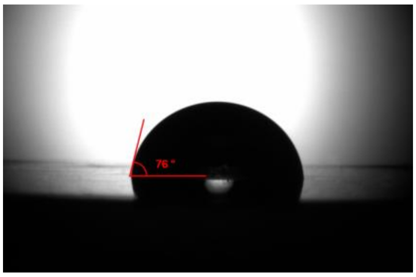

Figure S9. The contact angle of the $CsPbBr_3$@FDTS NCs.

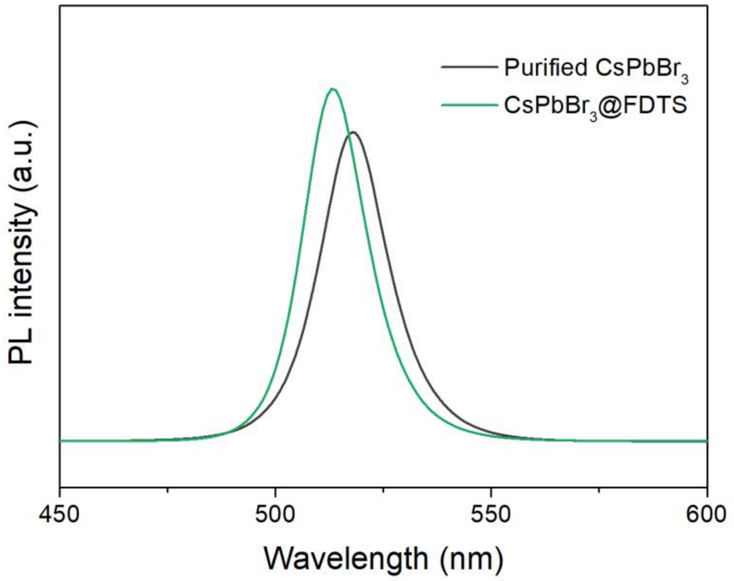

Figure S10. The PL signal of the purified $CsPbBr_3$ and $CsPbBr_3$@FDTS.

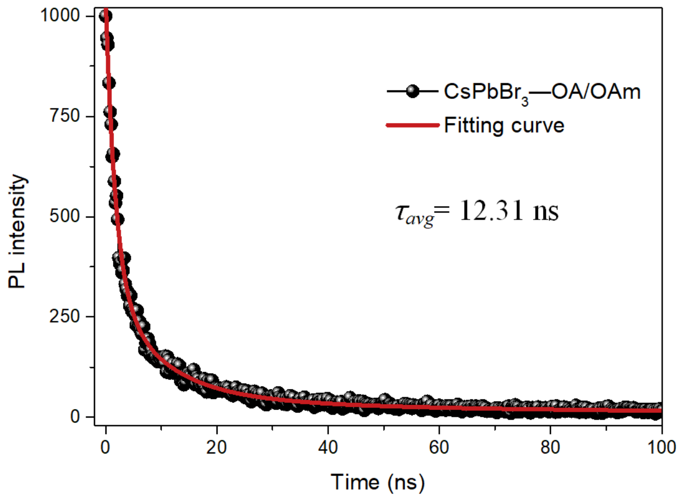

Figure S11. Time-resolved PL decay and fitted curve of $CsPbBr_3$ OA/OAm.

Table S1. Fitting parameters and average lifetimes of $CsPbBr_3$ NCs.

	A_1	τ_1 [ns]	A_2	τ_2 [ns]	T_{avg}	Adj.R
Purified $CsPbBr_3$	914.59767	2.03613	242.87884	14.9105	10.53841	0.99318
$CsPbBr_3$@FDTS	892.95127	3.66856	289.18005	29.92247	22.71273	0.99291
$CsPbBr_3$ OA/OAm	951.36362	3.2841	274.24197	18.01661	12.30945	0.99513

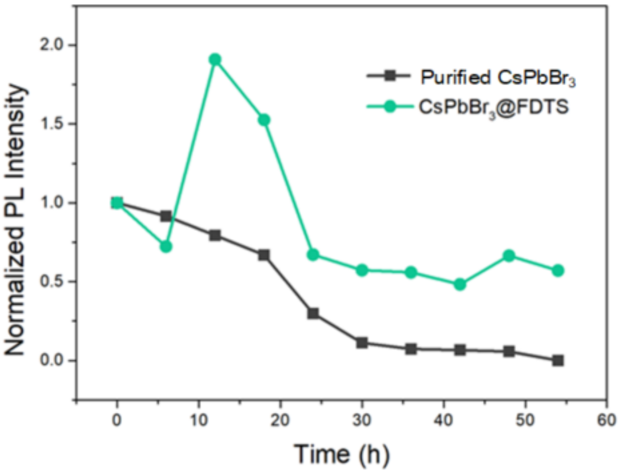

Figure S12. Time-dependent PL intensity of the perovskite NCs under harsh conditions (85 °C high temperature and 85% relative humidity).

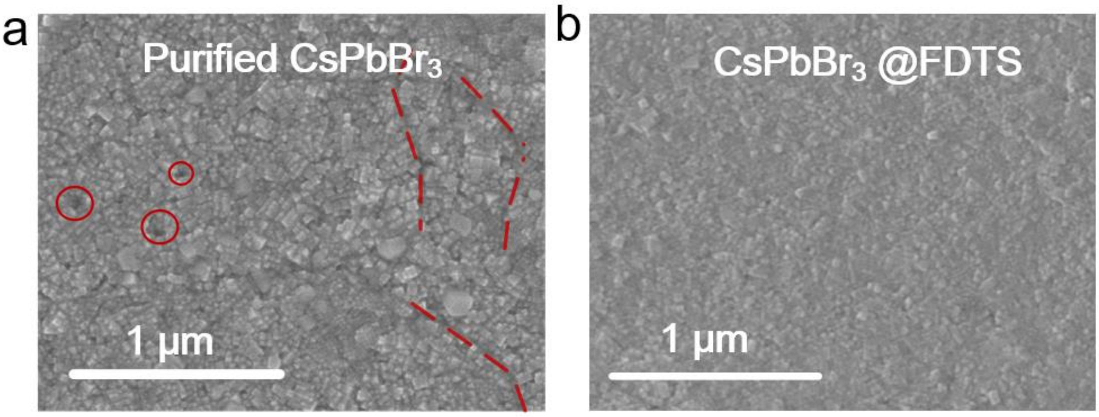

Figure S13. SEM images: (a) purified $CsPbBr_3$ and (b) $CsPbBr_3$@FDTS film.

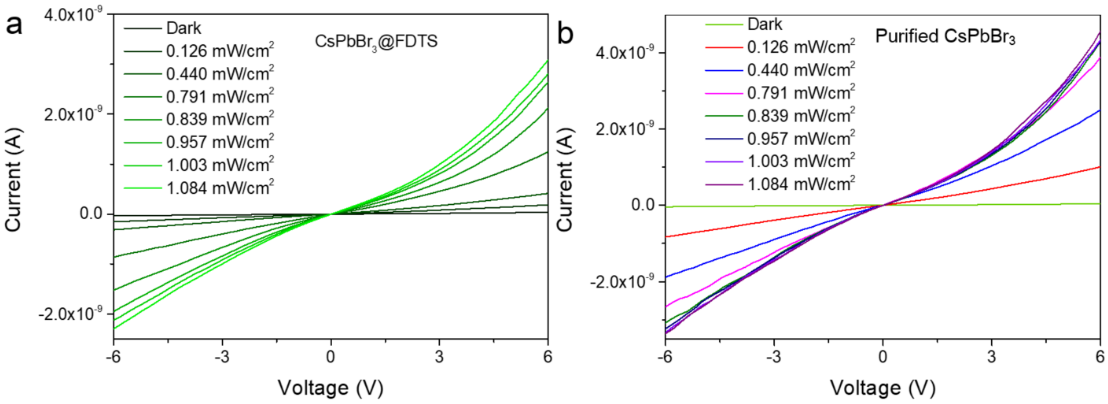

Figure S14. I–V curves: (a) purified $CsPbBr_3$ and (b) $CsPbBr_3$@FDTS photodetectors under different illumination intensities at a bias of 5 V.

Table S2. The performance of detectors fabricated by $CsPbBr_3$@FDTS with different parameters.

Factor	Experiment parameter	Responsivity (mA/W)	Detectivity ($\times 10^{10}$ Jones)
present	0.4 mmol CsBr, $PbBr_2$;<br>OAm (0.1 mL) and OA (1 mL);<br>FDTS dispersion (1‰)	13.60	7.75
NC size (bigger)	0.4 mmol CsBr, $PbBr_2$;<br>OAm (0.05 mL) and OA (0.5 mL);<br>FDTS dispersion (1‰)	16.66	4.77
NC size (smaller)	0.4 mmol CsBr, $PbBr_2$;<br>OAm (0.4 mL) and OA (1 mL);<br>FDTS dispersion (1‰)	12.45	7.15
FDTS-to-NCs ratio	0.4 mmol CsBr, $PbBr_2$;<br>OAm (0.1 mL) and OA (1 mL);<br>FDTS dispersion (0.5‰)	22.92	6.02
	0.4 mmol CsBr, $PbBr_2$;<br>OAm (0.1 mL) and OA (1 mL);<br>FDTS dispersion (2‰)	8.05	3.32
	0.4 mmol CsBr, $PbBr_2$;<br>OAm (0.1 mL) and OA (1 mL);<br>FDTS dispersion (5‰)	5.36	2.47
	0.4 mmol CsBr, $PbBr_2$;<br>OAm (0.1 mL) and OA (1 mL);<br>FDTS dispersion (1%)	3.48	1.88
	0.4 mmol CsBr, $PbBr_2$;<br>OAm (0.1 mL) and OA (1 mL);<br>FDTS dispersion (2%)	2.62	1.48
	0.4 mmol CsBr, $PbBr_2$;<br>OAm (0.1 mL) and OA (1 mL);<br>FDTS dispersion (5%)	-	-

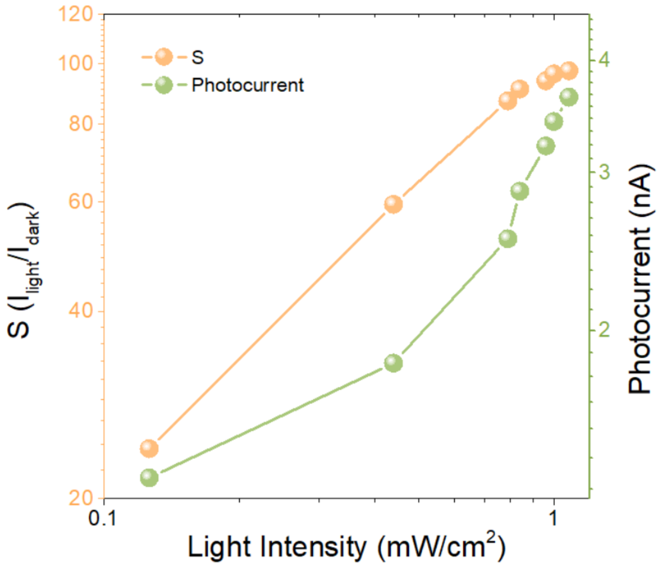

Figure S15. Photocurrent and switch ratio of the purified $CsPbBr_3$ NCs devices as a function of light intensity at a bias of 5 V.

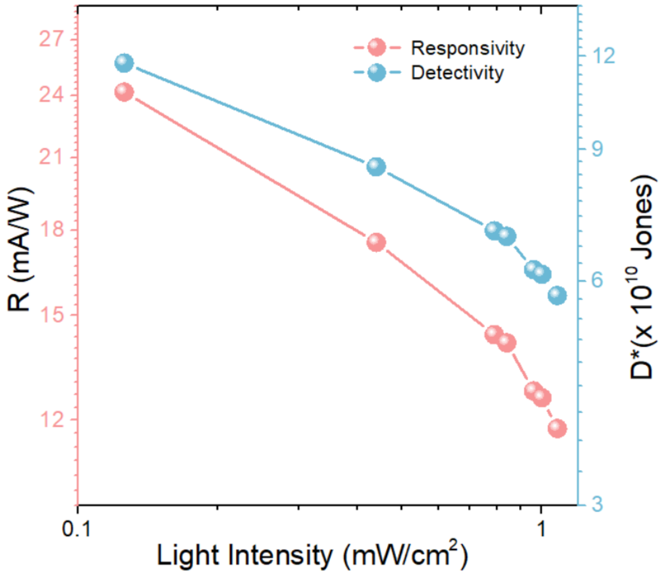

Figure S16. Responsivity and specific detectivity of the purified $CsPbBr_3$ NCs devices as a function of the light intensity at a bias of 5 V.

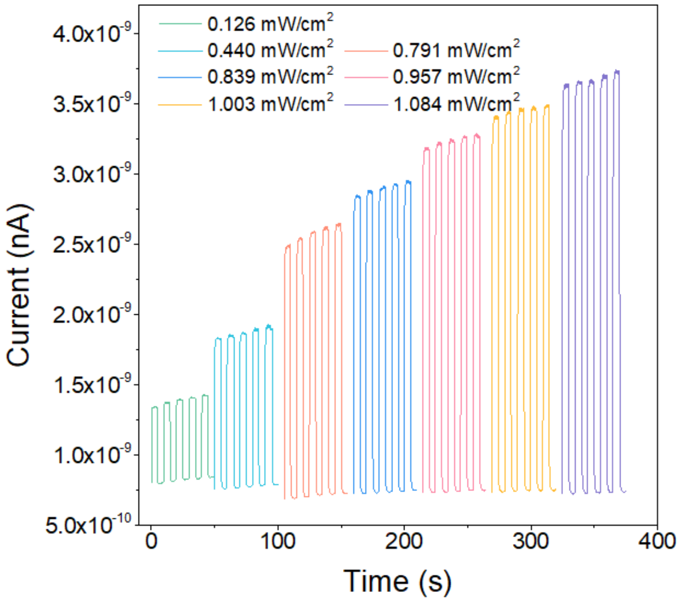

Figure S17. Current−time characteristics of the purified $CsPbBr_3$ NCs devices under different illumination intensities at a bias of 5 V.

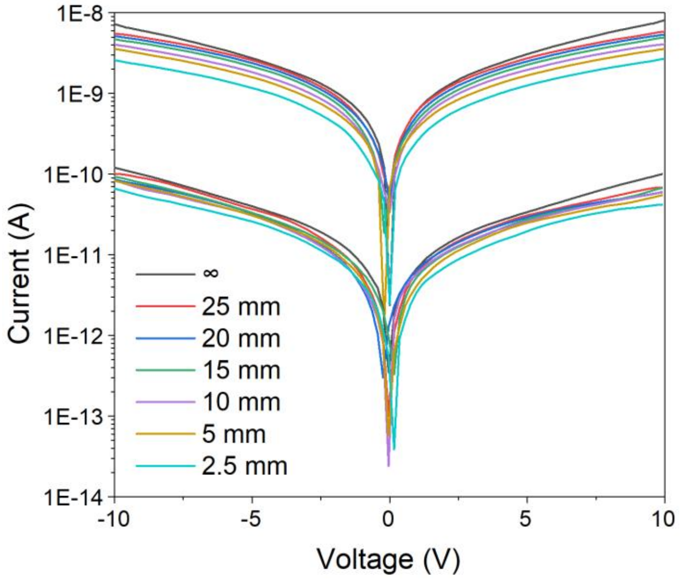

Figure S18. I–V curves of the purified $CsPbBr_3$ photodetectors under different bending radii.

Table S3. Summary of the photoresponse performance figure-of-merits of representative photodetectors

	**Sensing material**	**Spectral region**	**Responsivity(A/W)**	**Detectivity (Jones)**	**Reference**
**Flexible detector**	$CsPbBr_3$@FDTS NCs	UV	13.60×10^{-3}	7.75×10^{10}	This work
	CdTe NC	UV	8.0×10^{-6}	/	[1]
	ZnO NC	UV	8.5×10^{-3}	/	[2]
	PbS QD	Visible-NIR	3.8×10^{-3}	/	[3]
	ZnO NW	UV	/	/	[4]
	HgTe QD	NIR	/	7.5×10^{10}	[5]
	Organic/polymer thin-film	Photo/Thermal	433×10^{-3}	/	[6]
**Perovskite**	$MAPbI_3$ NW	UV–Visible	$\approx3.5 \times 10^{-2}$	$\approx10^{10}$	[7]
	$CsPbBr_3$ films	UV	0.24×10^{-3}	$\approx10^{10}$	[8]
	$MAPbI_3$ NW	UV	/	4.16×10^{12}	[9]
	$MAPbI_3$ NCs	UV	10.04×10^{-3}	4.56×10^{8}	[10]
	$CsPbI_3$ NCs	UV–Visible	30×10^{-3}	1.8×10^{11}	[11]
	$CsPbBr_3$ NCs	UV	1.72	1.76×10^{7}	[12]
	$CsPbX_3$ NCs	visible	/	/	[13]
	$CsPbBr_3$ NWs	UV	0.19	/	[14]
	ZnO NRs/$CsPbBr_3$ QDs	UV	0.14	/	[15]
	$CsPbBr_3$ NCs	visible	4.71×10^{-3}	4.56×10^{8}	[16]
	$CsPbX_3$ QDs	UV	32×10^{-3}	/	[17]
	$CsPbBr_3$ NCs	UV	2.2×10^{3}	1.1×10^{9}	[18]

	$CsPbBr_3$	UV–Visible	0.18	6.1×10^{10}	[19]
	$MAPbI_3$ NW	Visible	5×10^{-3}	/	[20]

(NC: nanocrystal; QD: quautum dot; NW: nanowire)

Reference

[1] K. Kwak, K. Cho, S. Kim, *Nanotechnology* **2011**, 22, 415204.

[2] J. Wu, L. Y. Lin, *Adv. Opt. Mater.* **2015**, 3, 1530.

[3] J. He, K. Qiao, L. Gao, H. Song, L. Hu, S. Jiang, J. Zhong, J. Tang, *ACS Photonics* **2014**, 1, 936.

[4] S. Bai, W. Wu, Y. Qin, N. Cui, D. J. Bayerl, X. Wang, *Adv. Funct. Mater.* **2011**, 21, 4464.

[5] X. Tang, M. M. Ackerman, G. Shen, P. Guyot-Sionnest, *Small* **2019**, 15, 1804920.

[6] X. Liu, Y. Guo, Y. Ma, H. Chen, Z. Mao, H. Wang, G. Yu, Y. Liu, *Adv. Mater.* **2014**, 26, 3631.

[7] L. Gu, M. M. Tavakoli, D. Zhang, Q. Zhang, A. Waleed, Y. Xiao, K.-H. Tsui, Y. Lin, L. Liao, J. Wang, Z. Fan, *Adv. Mater.* **2016**, 28, 9713.

[8] T. Zhang, F. Wang, P. Zhang, Y. Wang, H. Chen, J. Li, J. Wu, L. Chen, Z. D. Chen, S. Li, *Nanoscale* **2019**, 11, 2871.

[9] D. Wu, H. Zhou, Z. Song, M. Zheng, R. Liu, X. Pan, H. Wan, J. Zhang, H. Wang, X. Li, H. Zeng, *ACS Nano* **2020**, 14, 2777.

[10] Y. Li, X. Xu, C. Wang, B. Ecker, J. Yang, J. Huang, Y. Gao, *J. Phys. Chem. C* **2017**, 121, 3904.

[11] K. M. Sim, A. Swarnkar, A. Nag, D. S. Chung, *Laser & Photonics Rev* **2018**, 12, 1700209.

[12] D. M. Jang, D. H. Kim, K. Park, J. Park, J. W. Lee, J. K. Song, *J. Phys. Chem. C* **2016**, 4, 10625.

[13] P. Ramasamy, D.-H. Lim, B. Kim, S.-H. Lee, M.-S. Lee, J.-S. Lee, *ChemComm* **2016**, 52, 2067.

[14] S. Pan, H. Zou, A. C. Wang, Z. Wang, J. Yu, C. Lan, Q. Liu, Z. L. Wang, T. Lian, J. Peng, Z. Lin, *Angew. Chem. Int. Ed. Engl.* **2020**, 59, 14942.

[15] H. Wang, P. Zhang, Z. Zang, *Appl. Phys. Lett.* **2020**, 116, 162103.

[16] Y. Dong, Y. Gu, Y. Zou, J. Song, L. Xu, J. Li, J. Xue, X. Li, H. Zeng, *Small* **2016**, 12, 5622.

[17] J. Lu, X. Sheng, G. Tong, Z. Yu, X. Sun, L. Yu, X. Xu, J. Wang, J. Xu, Y. Shi, K. Chen, *Adv. Mater.* **2017**, 29, 1700400.

[18] Y. Che, X. Cao, Y. Zhang, J. Yao, *Opt. Mater.* **2020**, 100, 109664.

[19] X. Li, D. Yu, F. Cao, Y. Gu, Y. Wei, Y. Wu, J. Song, H. Zeng, *Adv. Funct. Mater.* **2016**, 26, 5903.

[20] E. Horváth, M. Spina, Z. Szekrényes, K. Kamarás, R. Gaal, D. Gachet, L. Forró, *Nano Lett.* **2014**, 14, 6761.
